# Inhibition of HIV Replication by Cyclic and Hairpin PNAs Targeting the HIV-1 TAR RNA Loop

**DOI:** 10.1155/2012/591025

**Published:** 2012-09-17

**Authors:** Gregory Upert, Audrey Di Giorgio, Alok Upadhyay, Dinesh Manvar, Nootan Pandey, Virendra N. Pandey, Nadia Patino

**Affiliations:** ^1^Institut de Chimie de Nice, UMR CNRS 7272, Université de Nice-Sophia Antipolis, 28 Avenue de Valrose, F06100 Nice, France; ^2^Department of Biochemistry and Molecular Biology, New Jersey Medical School, University of Medicine and Dentistry of New Jersey, 185 South Orange Avenue, Newark, NJ 07103, USA

## Abstract

Human immunodeficiency virus-1 (HIV-1) replication and gene expression entails specific interaction of the viral protein Tat with its transactivation responsive element (TAR), to form a highly stable stem-bulge-loop structure. Previously, we described triphenylphosphonium (TPP) cation-based vectors that efficiently deliver nucleotide analogs (PNAs) into the cytoplasm of cells. In particular, we showed that the TPP conjugate of a linear 16-mer PNA targeting the apical stem-loop region of TAR impedes Tat-mediated transactivation of the HIV-1 LTR *in vitro* and also in cell culture systems. In this communication, we conjugated TPP to cyclic and hairpin PNAs targeting the loop region of HIV-1 TAR and evaluated their antiviral efficacy in a cell culture system. We found that TPP-cyclic PNAs containing only 8 residues, showed higher antiviral potency compared to hairpin PNAs of 12 or 16 residues. We further noted that the TPP-conjugates of the 8-mer cyclic PNA as well as the 16-mer linear PNA displayed similar antiviral efficacy. However, cyclic PNAs were shown to be highly specific to their target sequences. This communication emphasizes on the importance of small constrained cyclic PNAs over both linear and hairpin structures for targeting biologically relevant RNA hairpins.

## 1. Introduction

**Figure 1:**
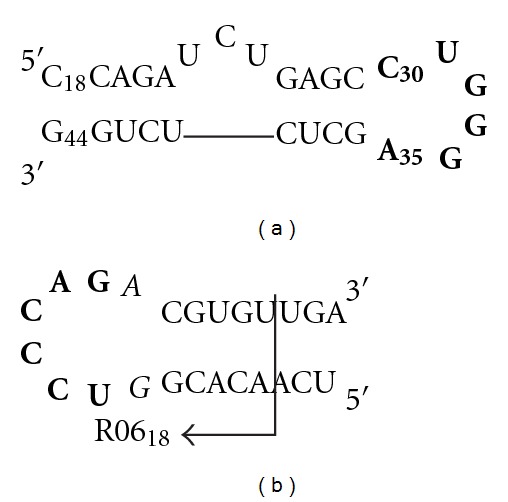
Sequence and secondary structure of (a) HIV-1 mini-TAR RNA, (b) R06_24_ and R06_18_ aptamers reported in this study. Bold bases indicate complementarity between aptamer and TAR loops. The crucial G and A residues flanking the R06 aptamers loop are in italics.

The transcriptional transactivation of the HIV-1 genome requires a specific interaction between the highly conserved TAR RNA hairpin fragment with the viral Tat protein and cellular factors (PTEFb-cyclin T1-CDK9 kinase complex). Both the six-nucleotide loop and the three-nucleotide bulge of TAR RNA (Figure 1(a)) are involved in the formation of this complex [1–3]. Therefore, molecules that can bind to the bulge or the loop of TAR are of great therapeutic interest, since disruption of the ternary complex formation leads to abortive mRNA synthesis and, consequently, to inhibition of viral replication.

During the last decade, a wide number of TAR ligands have been described [4, 5]. Among them, one can cite R06 aptamers (such as R06_24_ or R06_18_, Figure 1(b)), which were identified initially by *in vitro* selection [6]. These aptamers are folded RNA stem-loop structures which recognize the mini-TAR fragment (Figure 1(a)) not only on the basis of sequence complementarity, as classical antisense oligomers, but also on the basis of the tertiary structure of their target. This leads to highly stable and specific loop-loop complexes, also called “kissing complexes.” The key features for the establishment of such complexes are the hairpin structure of R06 aptamers as well as the octameric loop constituted by the 5′-UCCCAG-3′ sequence complementary to the TAR hexaloop, flanked by a G and a A residues. Although these two G/A residues are not directly involved in the loop-loop interaction, they were shown to be crucial for the formation of a stable kissing complex [7–10].

In a cellular compartment, RNA aptamers are rapidly degraded by nucleases, limiting their potential as therapeutic agents. Thus, several chemically-modified R06 derivatives were prepared with the view of improving both the pharmacological properties and TAR affinity. N3- > P5 phosphoramidate [11, 12], 2-O-methyl RNA [13, 14], and some hexitol nucleic acids (HNA)/RNA mixmers [15] were shown to display an improved nuclease resistance while maintaining a similar TAR-binding constant. TAR-binding properties of R06 analogs containing LNA residues were also studied [10, 16–19]. While the fully modified LNA version of R06 proved to be a poor TAR ligand, some chimeric LNA/DNA, and LNA/2′-OMe RNA aptamers displayed binding properties of interest. However, the identification of such chimeric aptamers is laborious, because it requires a systematic screening of all possible combinations, as no rule dictates the number and positions at which LNA nucleotides have to be incorporated to allow a strong loop-loop interaction.

Concerning the biological activity of these aptamer analogs, although some of them were shown to inhibit specifically Tat-mediated transcription in cell-free assays [12, 13, 15, 20, 21] or in cell assays when transfected with cationic lipids [17], none of them was evaluated as anti-HIV agents. However, it was shown that, when expressed endogenously in HeLa cells, the RNA aptamer R06 was able to inhibit HIV replication [22], highlighting the antiviral potential of nuclease resistant molecules that recognize the TAR loop through both their primary sequence and their tertiary structure.

**Figure 2:**
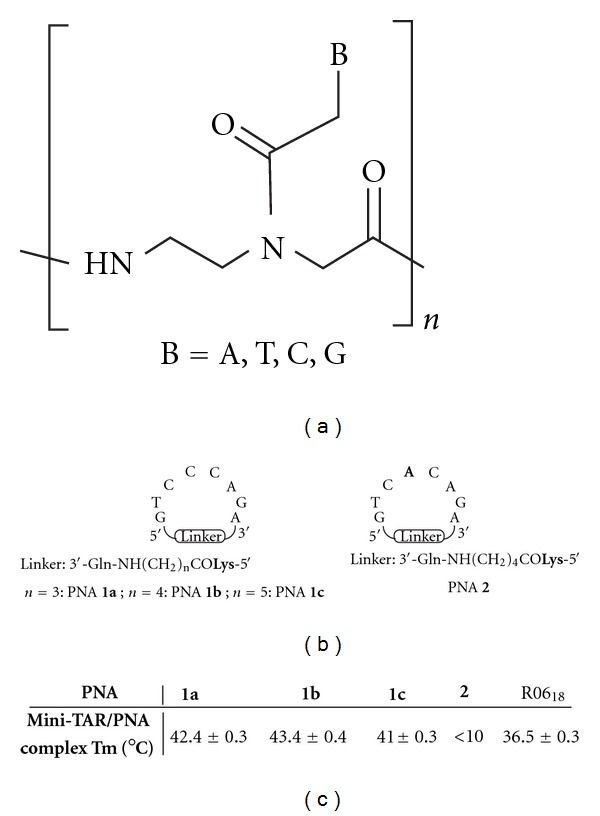
General structures of: (a) PNAs (b) cyclic PNAs **1a–c**, **2**; in bold, mismatched residue; (c) Melting temperatures of cyclic PNAs **1a–c**, **2**/TAR and R06_18_ aptamer/TAR complexes, obtained from [23].

Based on these results, we previously devised small synthetic constrained structures derived from the R06 aptamer derivatives, and reported that they were able to interact with the TAR loop through “kissing-like” complexes of high affinity [23]. These structures are constituted by an octameric PNA (Figure 2) 5′-GTCCCAGA-3′ sequence identical to the one found in R06 aptamers, head-to-tail cyclized *via* polyamide linkers of different length (**1a–c**, Figure 2(b)). We chose to introduce PNAs as RNA mimics since they are highly stable in biological media and they hybridize strongly with complementary RNA sequences [24]. A limitation to their *in vivo* applications is their poor ability to cross cell membranes. Thus, a lysine residue was incorporated in cyclic PNAs to allow their subsequent conjugation with cell penetrating moieties [25–27].

**Figure 3:**
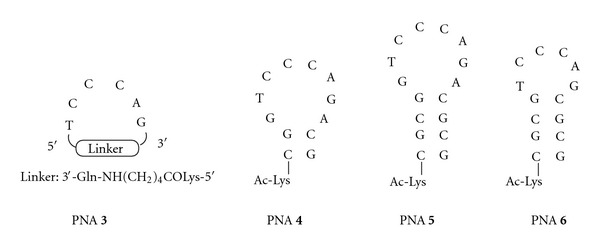
General structures of cyclic and hairpin PNAs synthesized in the present study.

**Figure 4:**
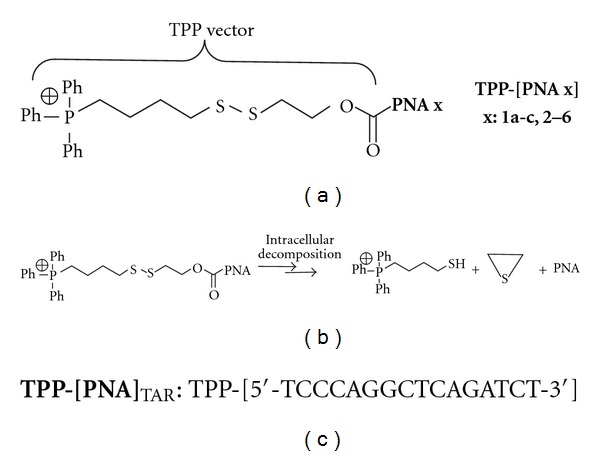
(a) Structures of the TPP conjugates of PNAs **1a–c**, **2–6**. (b) Schematic representation of the intracellular degradation mode of TPP-PNA conjugates (from [28]). (c) Structure of the reference compound used in this study (16-mer antisense TPP-[PNA]_TAR_).

Here, we report the synthesis of cyclic PNAs (**1–3**, Figures 2(b) and 3) and hairpin PNAs (**4–6**, Figure 3) conjugated to a triphenylphosphonium (TPP)-based cell penetrating vector (Figure 4(a)). This vector is constituted by a TPP lipophilic cation capable of transporting PNAs across the lipid bilayer [28, 29], bound *via* a disulfide bridge, to a mercaptoethoxycarbonyl moiety connected to the PNA. Intracellularly, the reduction of the disulfide bond leads to a spontaneous decomposition that releases the PNAs (Figure 4(b)) [30]. The cyclic PNAs exhibited potent anti HIV-1 activity in comparison to other derivatives, confirming the therapeutic potency of these conjugates.

## 2. Material and Methods

### 2.1. General Methods

Reagents and solvents were obtained from commercial sources and used without further purification unless indicated. Analytical HPLC chromatograms were obtained using an HP1100 UV detector set at 260 nm and a Beckman Ultrasphere RP-C18 (5 *μ*m) column at 55°C. Purifications using semipreparative HPLC were done on the same instrument using a Phenomenex Jupiter column RP-C18 (5 *μ*m). Elution solvent A was water (0.1% TFA); elution solvent B was acetonitrile (0.1% TFA). ESI mass spectra were recorded with a Bruker Esquire 3000 plus. Concentrations of cyclic PNAs, hairpin PNAs, and TPP conjugates were determined by UV spectroscopy, using the usual extinction coefficients [31]. The mini-TAR RNA fragment used for thermal denaturation studies was purchased at Dharmacon Inc. (Lafayette, USA). Thermal denaturations of mini-TAR/PNAs complexes were carried out on a Varian Cary 300 Scan spectrophotometer.

### 2.2. Chemistry

#### 2.2.1. Synthesis of Cyclic PNA 3 and Hairpin PNAs 4–6

These PNAs were synthesized in Merrifield vessels on MBHA resin (100–200 mesh, 0.63 mmol/g, Merck Schuchardt OHG, Hohenbrunn, Germany), on a 100-*μ*mol scale. Elongation was carried out starting from Boc/Z protected PNA monomers, using HBTU as the coupling reagent, and NMP as solvent. Compound 3 was synthesized as previously described for cyclic PNAs **1a–c** and **2** [23]. The lysine residue at the 5′-end of hairpin PNAs **4–6** was introduced after elongation, by means of Boc-Lys(2-Cl-Z)-OH and HBTU as the activator. Acetylation of the lysine residue was performed after Boc deprotection (TFA/TIS 10%, 4 mL for 15 min), using an Ac_2_O/pyridine/NMP 1/1/8 v/v/v solution (2 × 4 mL for 15 min). Compounds **4–6** were deprotected and cleaved from the resin using a TFMSA/TFA/TIS solution (1 : 8 : 1) for 4 h, then precipitated in cold anhydrous diethyl ether. Crude products were isolated by centrifugation (3,000 min^−1^, −4°C), washed twice with diethyl ether (10 mL), and purified by semipreparative HPLC using the following method: 55°C, A/B 100/0 for 7 min, then from 100/0 to 50/50 for 45 min, with a flow rate of 2 mL/min.

#### 2.2.2. c(Lys-TCCCAG-Gln)_*n*  =  4_ (PNA 3)

HPLC (A/B 100/0 for 7 min, then from 100/0 at 50/50 for 45 min):  *tR * = 17.5 min; MS (ESI, positive mode): (calculated for C_79_H_108_N_38_O_22_ : 1941.9) *m*/*z* = 1943.3 (M+H)^+^, 971.9 (M+2H)^2+^/2, 648.5 (M+3H)^3+^/3.

#### 2.2.3. Ac-Lys-CGGTCCCAGACG-NH_2_ (PNA 4)

HPLC (A/B 100/0 for 7 min, then from 100/0 at 50/50 for 45 min):  *tR* = 21.3 min; MS (ESI, positive mode): (calculated for C_136_H_175_N_73_O_37_ : 3424.41) *m*/*z* = 1713.4 (M+2H)^2+^/2, 1142.9 (M+3H)^3+^/3, 857.5 (M+4H)^4+^/4, 686.5 (M+5H)^5+^/5.

#### 2.2.4. Ac-Lys-CGCGGTCCCAGACGCG-NH_2_ (PNA 5)

HPLC (A/B 100/0 for 7 min, then from 100/0 at 50/50 for 45 min): *tR* = 23.5 min; MS (ESI, positive mode): (calculated for C_178_H_227_N_97_O_49_ : 4508.83), *m*/*z* = 2255.5 (M+2H)^2+^/2 1504.4 (M+3H)^3+^/3, 1128.7 (M+4H)^4+^/4, 903.3 (M+5H)^5+^/5, 753.2 (M+6H)^6+^/6.

#### 2.2.5. Ac-Lys-CGTCCCAGCG-NH_2_ (PNA 6)

HPLC (A/B 100/0 for 7 min, then from 100/0 at 50/50 for 45 min):  *tR * = 19.9 min; MS (ESI, positive mode): (calculated for C_113_H_148_N_60_O_32_ : 2858.8), *m*/*z* = 1430.7 (M+2H)^2+^/2, 953.5 (M+3H)^3+^/3, 715.5 (M+4H)^4+^/4.

#### 2.2.6. Synthesis of TPP-[PNA] Conjugates

An NMP solution containing TEA (10 eq.), NaN_3_ (4 eq.) and compound 7 (4 eq.) was added at 0°C to pure PNAs **1a–c**, **2–6** (1 eq.) dissolved in a NMP/DMSO 1/1 v/v solution to obtain a 5 mM final concentration. The mixture was stirred for 30 min at 0°C, then for 12 h at room temperature. Cold diethyl ether (10-fold volume) was added to the mixture. Crude products were isolated by centrifugation (3,000 min^−1^, −4°C) and washed twice with diethyl ether (10-fold volume). These products were further purified by a semipreparative HPLC method: A/B 100/0 for 7 min, then from 100/0 to 20/80 for 45 min, at 55°C, with a flow rate of 2 mL/min, to give, after lyophilization, TPP-[PNAs **1a–c**, **2–6**].

#### 2.2.7. TPP-[PNA 1a]

HPLC (1 mL/min, A/B 100/0 for 7 min, then from 100/0 to 20/80 in 45 min):  *tR * = 21.6 min; MS (ESI, positive mode): (calculated for C_126_H_159_N_51_O_29_PS_2_^+^ : 2946.17) *m*/*z* = 1473.4 (M+H)^2+^/2 982.4 (M+2H)^3+^/3, 736.9 (M+3H)^4+^/4, 589.7 (M+4H)^5+^/5.

#### 2.2.8. TPP-[PNA 1b]

HPLC (1 mL/min, A/B 100/0 for 7 min, then from 100/0 to 20/80 in 45 min):  *tR * = 23.8 min; MS (ESI, positive mode): (calculated for C_127_H_161_N_51_O_29_PS_2_^+^ : 2960.19) *m*/*z* = 988.0 (M+2H)^3+^/3, 741.3 (M+3H)^4+^/4.

#### 2.2.9. TPP-[PNA 1c]

HPLC (1 mL/min, A/B 100/0 for 7 min, then from 100/0 to 20/80 in 45 min):  *tR * = 22.4 min; MS (ESI, positive mode): (calculated for C_128_H_163_N_51_O_29_PS_2_^+^ : 2974.21) *m*/*z* = 992.6 (M+2H)^3+^/3, 744.7 (M+3H)^4+^/4, 596.1 (M+4H)^5+^/5.

#### 2.2.10. TPP-[PNA 2]

HPLC (1 mL/min, A/B from 100/0 to 20/80 in 30 min):  *tR * = 13.2 min; MS (ESI, positive mode): (calculated for C_127_H_160_N_54_O_28_PS_2_^+^ : 2985.20) *m*/*z* = 1505.4 (M+Na)^2+^/2.

#### 2.2.11. TPP-[PNA 3]

HPLC (1 mL/min, A/B from 100/0 to 20/80 in 30 min):  *tR * = 12.4 min; MS (ESI, positive mode): (calculated for C_104_H_134_N_38_O_24_PS_2_^+^ : 2395.52) *m*/*z* = 1209.23 (M+Na)^2+^/2.

#### 2.2.12. TPP-[PNA 4]

HPLC (1 mL/min, A/B from 100/0 to 20/80 in 30 min):  *tR * = 12.7 min; MS (ESI, positive mode): (calculated for C_161_H_202_N_73_O_39_PS_2_^+^ : 3878.52), *m*/*z* = 1939.5 (M+H)^2+^/2, 1293.6 (M+2H)^3+^/3, 970.9 (M+3H)^4+^/4.

#### 2.2.13. TPP-[PNA 5]

HPLC (1 mL/min, from A/B 100/0 to 20/80 in 30 min):  *tR * = 12.8 min; MS (ESI, positive mode): (calculated for C_202_H_253_N_98_O_51_PS_2_^+^ : 4962.95) *m*/*z* = 1654.3 (M+2H)^3+^/3, 1241.5 (M+3H)^4+^/4, 994.0 (M+4H)^5+^/5.

#### 2.2.14. TPP-[PNA 6]

HPLC (1 mL/min, from A/B 100/0 to 20/80 in 30 min):  *tR * = 12.8 min; MS (ESI, positive mode): (calculated for C_138_H_174_N_60_O_34_PS_2_^+^ : 3312.32) *m*/*z* = 1656.1 (M+H)^2+^/2, 1105.2 (M+2H)^3+^/3.

### 2.3. Thermal Denaturation Studies

One nmol of mini-TAR was solubilized in 250 *μ*L (4 *μ*M concentration) of *R* buffer solution at pH 7.3, that buffer containing cacodylate (20 mM), NaCl (20 mM), KCl (140 mM), and MgCl_2_ (0.3 mM). The solution was heated at 90°C for 2 min, immediately cooled at 4°C, and maintained at this temperature for 10 min, then kept at 20°C. For preparing hairpin PNAs **4–6**, a solution of each compound in *R* buffer (4 *μ*M) was heated for 3 min at 95°C, then cooled to 20°C with a rate of 0.5°C/min [31]. Individual compounds **3**, **4–6** and mini-TAR in *R* buffer (2 *μ*M final concentration of each) were mixed, then incubated at 5°C for 1 h. Thermal denaturation was generated by increasing the temperature from 5°C, to 90°C at 0.4°C/min, then followed by UV absorption (260 nm). Melting temperatures (*T*_m_) were determined as the maximum of the first derivative of the melting curves.

### 2.4. Transfection and Production of HIV-1 Virions

For production of highly infectious pseudotyped HIV-1 virions, 293T cells grown in complete Dulbecco's modified Eagle's medium (DMEM) were cotransfected with pHIV-1JR-CSF-lucenv(−) and pVSV-G, using a calcium phosphate transfection system (Invitrogen Carlsbad, CA, USA) [32, 33]. The culture supernatant was saved at 24, 48, and 72 h after transfection, then pooled and analyzed for p24 antigen using the ELISA p24 antigen kit (ZeptomMetrix, Buffalo, NY, USA). The pseudotyped HIV-1 virions were then isolated from the culture supernatant by filtration through a 0.45 *μ*m pore size PVDF membrane (Millipore Bedford, MA, USA) and then by ultracentrifugation at 70,000 g for 45 min. The viral pellet was resuspended in complete Dulbecco's medium and stored at −80°C.

### 2.5. Anti-HIV-1 Activity in CEM Cells

CEM CD4+ lymphocytes 12D7 were grown in RPMI-1640 medium supplemented with 10% fetal calf serum and 4 mM L-glutamine at 37°C in 5% CO_2_ containing humidified air [34]. Early-to mid-log-phase cells were harvested and washed with an equal volume of PBS without Ca^2+^ and Mg^2+^. Approximately 10^6^ cells suspended in 250 *μ*L of RPMI-1640 medium were incubated with pseudovirions (equivalent to 25 ng of p24) by gentle rocking for 2 h in the presence of polybrene (10 *μ*g/mL). The infected cells were washed with PBS and resuspended in 1 mL of complete RPMI medium in a 24-well plate containing increasing amounts of individual TPP-[PNAs **1a–c**, **2–6**] (0.5 *μ*M–5 *μ*M). After 48 h, the cells were harvested, washed with PBS, and lysed in 1 × passive lysis buffer (Promega) with gentle shaking on a rocker for 15 min at room temperature. The lysed cells were centrifuged at 15,000 rpm for 15 min and an aliquot of the clear lysate was added to 100 *μ*L luciferase assay reporter (Promega) in 96-well plate Fluotrac 200 (Greiner Labortechnik, Germany). The luciferase activity was measured on a Packard Top Count Luminometer. The total light unit was normalized by total protein content in the cell lysate. Total protein was quantified using the DC protein assay kit (Bio-Rad Laboratories, Hercules, CA, USA). Median dose effects (IC_50_) for individual TPP-[PNAs **1a–c**, **2–6**] were determined using CalcuSyn software (Biosoft, Cambridge, UK) [35, 36].

## 3. Results and Discussion

Previously, we have shown that cyclic PNAs **1a–c** tightly bind to TAR (Figure 2(c)), with a higher affinity than that of a R06 aptamer (R06_18_, Figure 1) and that they were highly specific for TAR despite the limited number of bases constituting them, since the introduction of a single mismatch in the PNA sequence was strongly deleterious for TAR binding. Indeed, compound **2** (Figure 2(b)), in which the C4 residue was replaced by an A4 residue, showed no affinity for TAR [23]. The first goal of the present study was to assess whether these PNA structures, which are cyclized in a covalent way, are more advantageous for targeting the TAR loop than hairpin structures in which the loop is not covalently closed. Although PNAs are among the best nucleic acid mimics, no PNA analogue of R06 aptamers has been reported so far. Thus, we have prepared hairpin PNAs (compounds **4** and **5**, Figure 3) containing the same octameric PNA sequence than in cyclic PNAs **1a–c**, closed by two and four G-C pairs, respectively, and measured their affinity for TAR. The second goal of this study was to determine whether, as for R06 aptamer derivatives, the G and A PNA residues flanking the loop sequence are necessary for the establishment of stable loop-loop complexes. For this purpose, we synthesized the G- and A-deleted cyclic PNA **3** and hairpin PNA **6** (Figure 3), and studied their interaction with TAR. Finally, in order to evaluate the ability of both cyclic (**1a–c** and **2-3**) and hairpin (**4–6**) PNAs to inhibit HIV replication in infected cells, we conjugated them to a cell-penetrating vector, *via* their lysine residue (Figure 4(a)). The vector chosen in this study is an intracytoplasmic biodegradable triphenylphosphonium (TPP)-based moiety, which was shown to allow the uptake and release of a “naked” PNA into cytoplasm (i.e., without any residual TPP moiety attached to PNAs, Figure 4(b)) [30]. For antiviral activity studies, a previously described TPP conjugate of a 16-mer PNA_TAR_ targeting the apical stem-loop of TAR was taken as a reference compound (Figure 4(c)) [30].

### 3.1. Chemistry

The synthesis of compounds **1a–c** and **2** was previously reported [23]. Cyclic PNA **3** was prepared following a solid-phase strategy *via* on-resin cyclization, using a glutamic acid-anchored MBHA resin, as for cyclic PNAs **1a–c** and **2**. Hairpin PNAs **4–6** were synthesized on a MBHA resin, using standard procedures. Briefly, the elongation was performed using Boc/Z-protected PNA monomers and HBTU as coupling reagent. The lysine residue at the 5′-end was introduced after elongation by means of Boc-Lys(2-Cl-Z)-OH and HBTU. After Boc cleavage under acidic conditions (TFA/TIS 10%), the *α*-amino group of the lysine residue was acetylated using an Ac_2_O/pyridine/NMP solution. Hairpin PNAs **4–6** were obtained after deprotection and cleavage from the resin using a TFMSA/TFA/TIS solution and purification by semipreparative HPLC. Their structures were confirmed by ESI-MS experiments (see experimental protocols in Supplementry Material available online at doi:10.1155/2012/591025).

The TPP-conjugates of cyclic and hairpin PNAs were obtained in almost quantitative yields from their corresponding cyclic **1a–c**, **2-3** and hairpin **4–6** precursors, in one step, using an excess of the key para-nitrophenyl carbonate reagent **7** in the presence of sodium azide, as previously described [28]. The TPP-conjugates were purified by semipreparative HPLC, with an RP-C18 column and water (0.1% TFA) and acetonitrile (0.1% TFA) as the elution solvents. Their structures were confirmed by ESI-MS experiments in which the corresponding spectra displayed characteristic (M+*n*H)^(*n*+1)+^/(*n*+1) peaks (*n* = 1 to 5) (see experimental protocols in Supplementry Material). 

### 3.2. Thermal Denaturation Study

The affinity of compounds **3–6** for the mini-TAR RNA fragment was evaluated by thermal denaturation monitored by UV absorption spectroscopy (*λ*_max⁡_ = 260 nm) in *R* buffer, as previously reported for cyclic PNAs **1a–c** and **2** and R06_18_ aptamer [23]. Melting temperatures (*T*_m_) of the TAR/cyclic PNA **3** and TAR/hairpin PNAs(**4–6**) complexes are summarized in Table 1, together with the melting temperatures of hairpin PNAs **4–6** alone (i.e., without TAR).

**Figure 5:**
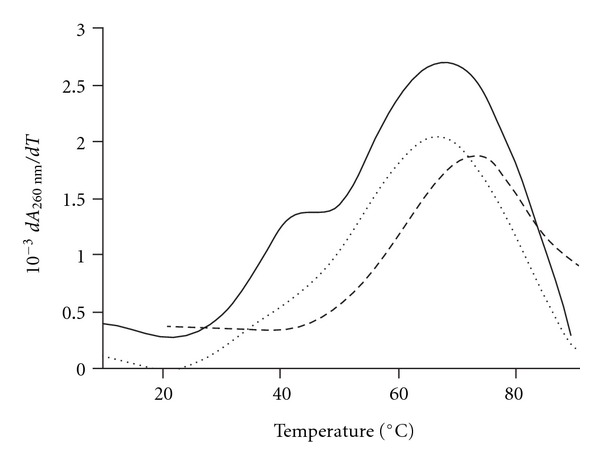
First derivative plot of melting curves followed by UV spectroscopy for hairpin PNA **4** alone (dashed line), TAR RNA alone (dotted line) and of hairpin PNA **4**/TAR complex (plain line).

Thermal denaturation studies of hairpin PNAs **4** and **5** alone exhibited a single transition at, respectively, 73.0°C and 84.0°C (Table 1), independently of PNA concentration, indicating that they fold to form highly stable hairpins [31]. The difference between their *T*_m_ values (Δ*T*_m_ = 11°C) reflects the higher stability of the double strand in PNA **5** than in PNA **4**, due to the presence of two additional canonical CG pairs in PNA **5** relative to PNA **4**. The melting profiles obtained with mixtures of mini-TAR and hairpin PNAs **4** or **5** displayed two transitions (e.g., see Figure 5). The highest one forms a broad peak, resulting from the overlapping of the two melting transitions concerning mini-TAR (*T*_m_ = 69.5°C) and hairpin PNAs **4** or **5**. The transition at the lowest temperature corresponds to the melting of the TAR/PNA **4** or **5** complex (45.1°C and 52.4°C, resp.). These values are significantly higher than those obtained with cyclic PNAs **1a–c**(Δ*T*_m_ ≥ +2 and ≥ 10°C, resp.), emphasizing the higher stability of the complexes formed with hairpin PNA **4** and **5** over cyclic PNAs. Nevertheless, both TAR/hairpin PNAs and TAR/cyclic PNAs complexes are more stable than TAR/RNA R06_18_ complex (*T*_m_ = 36.5°C) and than most of TAR/R06_24_ analogues ones, analyzed in the same conditions (for NP-DNA [11], 2′-OMe RNA [13] and HNA/RNA [15] R06_24_ derivatives: (*T*_m_ *≈* 30°C; for the best LNA/DNA R06_24_ derivative: (*T*_m_ > 40°C [16]).

Concerning the 5′-G and 3′-A deleted cyclic PNA **3**, the thermal denaturation study clearly demonstrates that it does not bind to TAR, highlighting the importance of the G and A flanking residues for the formation of a stable complex between TAR and cyclic PNAs. Conversely, the 5′-G and 3′-A deleted hairpin PNA **6** is able to smoothly interact with TAR. However, the melting temperature of the corresponding complex (38.4°C) is higher than the melting temperature of the hairpin PNA **6** itself (29.8°C). Thus, it is likely that the formation of the complex with TAR occurs, at least in part, on the unfolded form of **6**.

It has been earlier shown for TAR/TAR RNA aptamer complexes that the presence of G and A loop closing residues is a key structural determinant conferring a high stability both to the RNA aptamer alone and the TAR/RNA aptamer complex. Substituting the GA pair by the AU one (or GC, CA…) decreases the *T*_m_ of both the RNA aptamer and the TAR/RNA aptamer complex by 17°C and 14°, respectively [9]. Comparing PNA 5 and 6 shows that the presence of the GA pair also leads to a drastic increase in the stability of both the PNA hairpin alone and the TAR/PNA complex (Δ*T*_m_ of 54° and 14°C, resp.). NMR [8] and molecular dynamics studies [9] have shown that the presence of these two residues increases the stability of both the aptamer and the kissing complex by increasing the stacking at the stem-loop junctions, *via* the stabilization of two hydrogen-bond base pairs located at these stem-loop junctions of the kissing complex: the intramolecular G-A pair itself, *via* hydrogen bonding of N1-N1 carbonyl-amino type, and the intermolecular A-U pair, *via* the classical Watson-Cricks network. By contrast, when the loop of the aptamer is closed by the classical AU pair, the very high tension in the loop causes the opening of both this AU intramolecular pair and of the intermolecular AU one, leading to a less stable kissing complex. It is possible that such events also occur in the case of PNA **4–6** hairpins and corresponding TAR complexes but for the moment, no proof supports this hypothesis.

It is also possible that larger loop size of PNA **4/5** with a nonhydrogen bonding GA pair may offer greater stability to stem region as compared to small loop size of PNA 6 with a hydrogen-bonding GT pair closing the loop. The smaller loop size may cause strain on the stability of the stem region.

### 3.3. HIV-1 Inhibition in Cell Culture by Anti-TAR PNA

**Figure 6:**
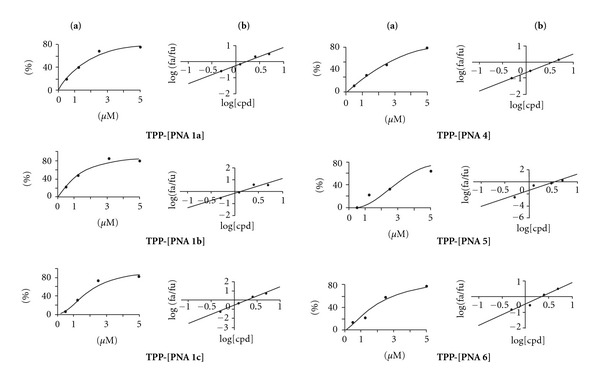
Dose-effect curves and median-effect plots for antiviral activity of TPP-conjugates. CEM cells were infected with VSV-G pseudotyped S1 strain of HIV-1 and grown in the presence of increasing concentrations of test conjugates. After 48 h, cells were harvested and assayed for luciferase expression using Luciferase assay kit (Promega). The effect of conjugate concentrations on the luciferase expression was analyzed using CalcuSyn software (Biosoft). The IC_50_ values, determined from the ratio of log of luciferase expression in treated cells (fa) and untreated cells (fu) as a function of the log concentration of conjugates was 1.0 > *M*; the linear correlation coefficient, *r*, ranged from 0.943 to 0.985. (a) Dose-effect curves for antiviral activity of TPP conjugates. (b) Median-effect plot.

In order to evaluate the *in vitro* antiviral efficacy, we incubated CEM CD4+ lymphocytes, infected with highly infectious VSV-G pseudotyped HIV-1 virions expressing the firefly luciferase reporter gene [30], with varying concentrations of individual TPP-[PNA **1a–c**, **2–6**]. Similar experiments were carried out with unconjugated PNAs **1a–c** and **4–6**. The TPP-conjugate of a 16-mer antisense [PNA]_TAR_ targeting the apical stem-loop of TAR was taken as a reference compound [30] (Figure 4(c)). We have previously showed that this compound inhibited HIV replication in infected cells at a micromolar concentration. To measure the effect of TPP-PNA conjugates on HIV-1 production in CEM cells, we monitored the expression of the firefly luciferase reporter gene, cloned instead of the nef gene in the HIV-1 virions. After 48 h incubation followed by cells lysis, the extracts were normalized for total protein content and analyzed for quantitative levels of luciferase expression. An arbitrary value of 100 was assigned to the luciferase activity obtained in infected cells in the absence of compounds; values relative to this control value were given to the other samples. Median dose effects (IC_50_) for individual compoundwere then determined using CalcuSyn software (Figure 6). As expected, no antiviral activity was detected for the unconjugated PNAs **1a–c** and **4–6**, probably as a consequence of their poor cellular permeation (data not shown). Median dose effects (IC_50_) obtained for individual TPP-[PNA **1a–c**, **2–6**] are summarized in Table 2.

In all cases, except TPP-conjugates of PNAs **2** and **3**, a substantial decrease in HIV-1 replication was observed as the concentration of TPP-conjugate was increased, the IC_50_ values ranging from 1.24 to 3.70 *μ*M (Table 2). The micromolar inhibitory effects measured for TPP conjugates of cyclic PNAs **1a–c** are very encouraging, because they are similar to those obtained with the heavier TPP-conjugate of the antisense 16-mer [PNA]_TAR_ (Figure 4(c)). As previously noticed for the complexes stability (*T*_m_ from to 41°C to 43.4°C, Table 1), the length of the linker closing cyclic PNAs **1a–c** (*n* = 3, 4, 5, Figure 2(b)) has little influence on the antiviral activity of their TPP conjugates (IC_50_ from 1.24 to 1.94 *μ*M). In addition, it appears that the antiviral activity of TPP-[PNA **1a–c**] conjugates is specific. Indeed, TPP-[PNA **2**] and TPP-[PNA **3**], which, respectively, derive from the mismatched cyclic PNA **2** and from the GA-deleted cyclic PNA **3**, have no effect on viral replication. These results, together with the fact that no interaction between mini-TAR and these two cyclic PNAs was detected, tend to further demonstrate that the inhibition of HIV replication by TPP-[PNAs **1a–c**] is related to the formation of cyclic PNAs **1a–c**/TAR complexes and thus, to the inhibition of the Tat/TAR/cellular factors complex formation.

Concerning TPP-hairpin PNAs (**4–6**) conjugates, it can be noted that the antiviral activity increases with the stability of the TAR/hairpin PNAs complexes [(TPP-[PNA **6**] *T*_m_ = 38.4°C, IC_50_ = 3.70 *μ*M < TPP-[PNA **4**] (*T*_m_ = 45.1°C, IC_50_ = 3.30 *μ*M < TPP-[PNA **5**] *T*_m_ = 52.4°C, IC_50_ = 2.10 *μ*M).] However, the complexes stability is not the only factor that explains biological activity, since cyclic PNAs **1a–c** conjugates (*T*_m_ values from 41°C to 43.4°C) are more effective for the inhibition of HIV replication in cells (IC_50_ 1.24–1.94 *μ*M) than the corresponding hairpin PNAs **4-5** conjugates.

Altogether, these results emphasize the advantage of cyclic PNA structures over hairpin ones for inhibiting HIV-1, through the targeting of the TAR RNA loop.

## 4. Conclusion

We demonstrated that the small cyclic PNAs targeting the HIV-1 TAR RNA loop inhibit viral replication when conjugated to a cell penetrating vector, as efficiently as do higher molecular weight compounds, such as hairpin PNAs or an anti-TAR 16-mer PNA antisense targeting both the stem and loop of TAR. Furthermore, despite their short PNA sequence, they are highly specific for their RNA target, since the introduction of a single mismatch in the PNA sequence is detrimental both for TAR binding and HIV inhibition. In addition, these results, combined with the high PNA stability in biological media, indicate that such cyclic compounds hold potential as new anti-HIV agents. On the other hand, these results emphasize the advantage of using small constrained cyclic structures over both linear antisense oligonucleotides and hairpin ones for targeting biologically relevant RNA hairpins.

## Supplementary Material

Supporting Information Available: A) HPLC and ESI MS spectra of PNA 3-6 and TPP-[PNA 1a-c, 2-6] conjugates (p. S2-S13). B) UV-monitored thermal denaturation: First-derivative melting curves of PAA 4-6 and PAA 4-6/TAR complexes (p. S14).

## Figures and Tables

**Table 1:** Melting temperatures (*T*_m__ _°C) of Mini-TAR/PNA complexes and of hairpin PNAs alone.

PNA	Mini-TAR/PNA complex *T*_m_ (°C)	Hairpin PNA alone *T*_m_(°C)
**3**	<10	—
**4**	45.1 ± 0.6	73.0 ± 0.3
**5**	52.4 ± 0.2	84.0 ± 0.5
**6**	38.4 ± 0.3	29.8 ± 0.6

Experiments were performed in R buffer (pH 7.3) containing cacodylate (20 mM), NaCl (20 mM), KCl (140 mM), and MgCl2 (0.3 mM). Individual PNAs and mini-TAR (2 *μ*M final concentration of each) were gently mixed and incubated at 5°C for 1 h. Thermal denaturation was achieved by increasing the temperature from 5°C to 90°C at the linear gradient of 0.4°C/min. The changes in UV absorption at 260 nm were monitored. Melting temperatures (*T*_m_) were determined as the maximum of the first derivative of the melting curves.

**Table 2:** IC_50_ values for TPP-[PNA] conjugates.

TPP-[PNA] conjugates	IC_50 _(*μ*M)	*r* ^a^	m^b^
TPP-[PNA **1a**]	1.63	0.983	1.15
TPP-[PNA **1b**]	1.24	0.953	1.23
TPP-[PNA **1c**]	1.94	0.985	1.99
TPP-[PNA **2**]	—^c^	—	—
TPP-[PNA **3**]	—^c^	—	—
TPP-[PNA **4**]	3.30	0.943	2.65
TPP-[PNA **5**]	2.10	0.974	1.39
TPP-[PNA **6**]	3.70	0.990	1.18
TPP-[PNA]_TAR_^d^	1.00	0.970	—

^a^Represents linear correlation coefficient of the median effect plot.

^b^Represents measurements of the sigmoidicity of the dose-effect curve.

^c^No inhibition up to 50 *μ*M.

^d^Obtained from [28].
