# Supplementary material for: Inhibition of HIV Replication by Cyclic and Hairpin PNAs Targeting the HIV-1 TAR RNA Loop
Source: J Nucleic Acids. 2012 Sep 17;2012:591025. doi: 10.1155/2012/591025 (PMC3457641; doi:10.1155/2012/591025)

Supporting Information

Inhibition of HIV replication by cyclic and hairpin PNAs targeting the HIV-1 TAR RNA loop

Gregory Upert, Audrey Di Giorgio, Alok Upadhyay, Dinesh Manvar, Nootan Pandey,Virendra N. Pandey* and Nadia Patino*

A - HPLC and ESI MS spectra of PNA (3-6) and TPP-[PNA 1a-c, 2-6] S2

B - Thermal denaturation followed by UV spectroscopy..S14

A - HPLC and ESI MS spectra of PNAs (3-6) and TPP-[PNA 1a-c, 2-6]

c(Lys-TCCCAG-Gln)$_{n=4}$ (PNA 3)

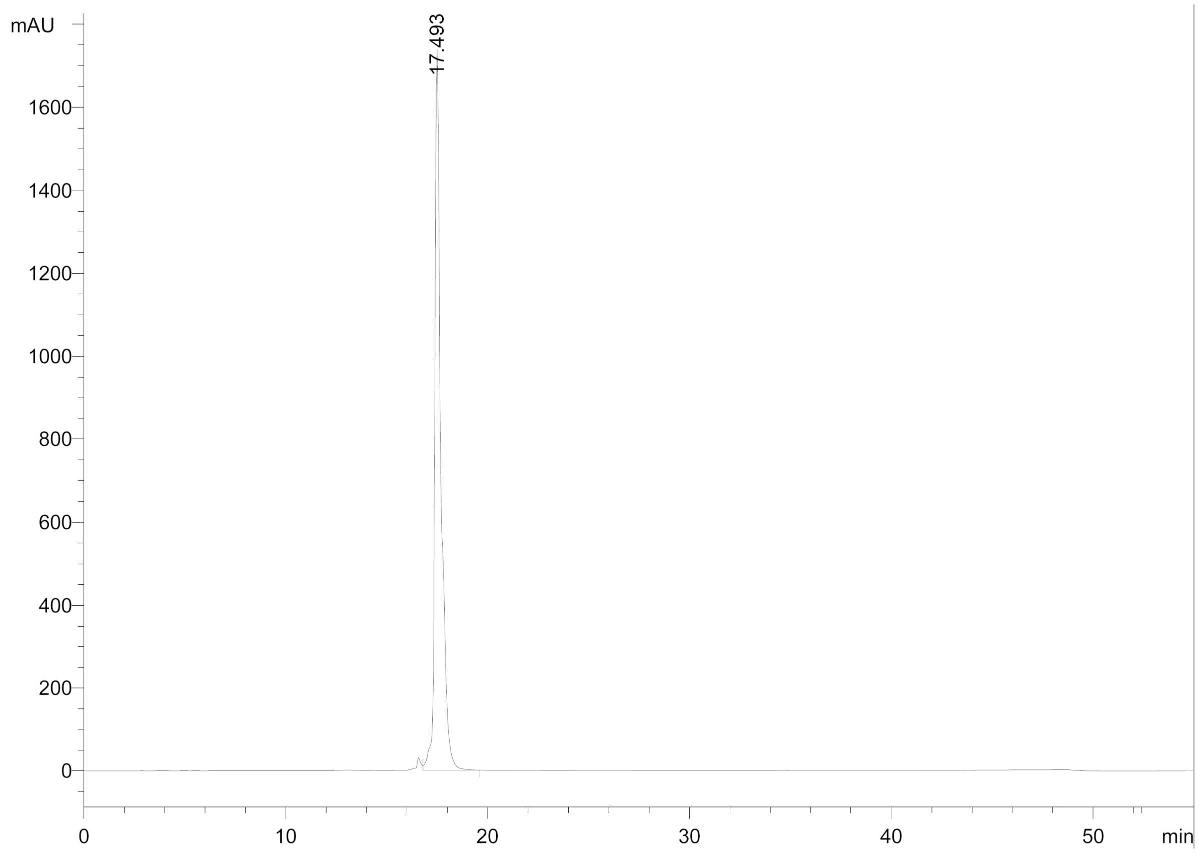

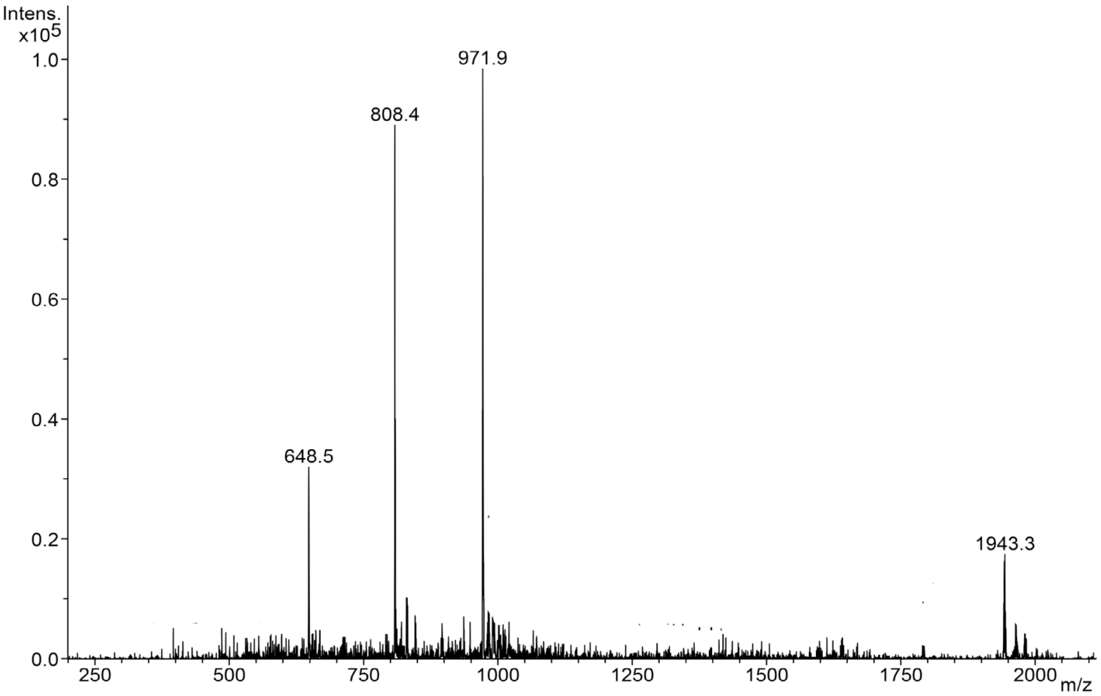

Ac-Lys-CGGTCCCAGACG-NH$_2$ (PNA 4)

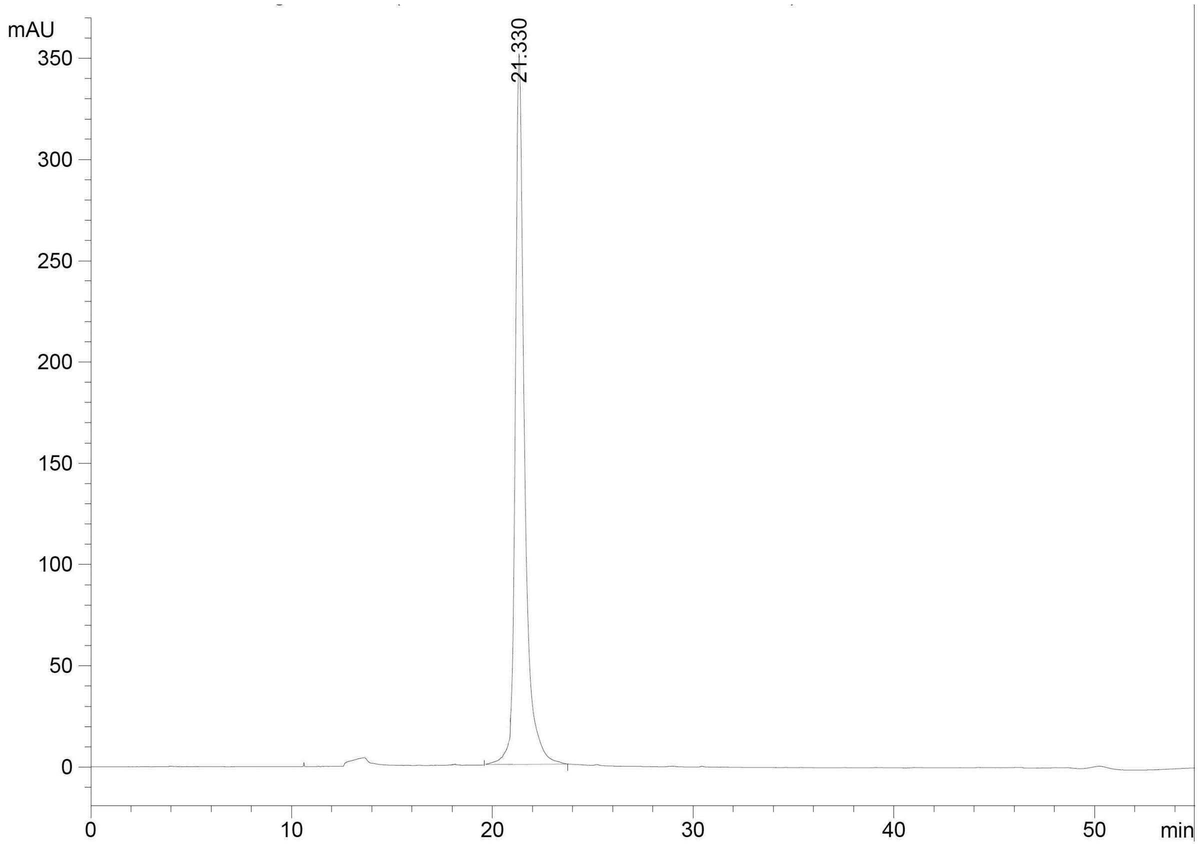

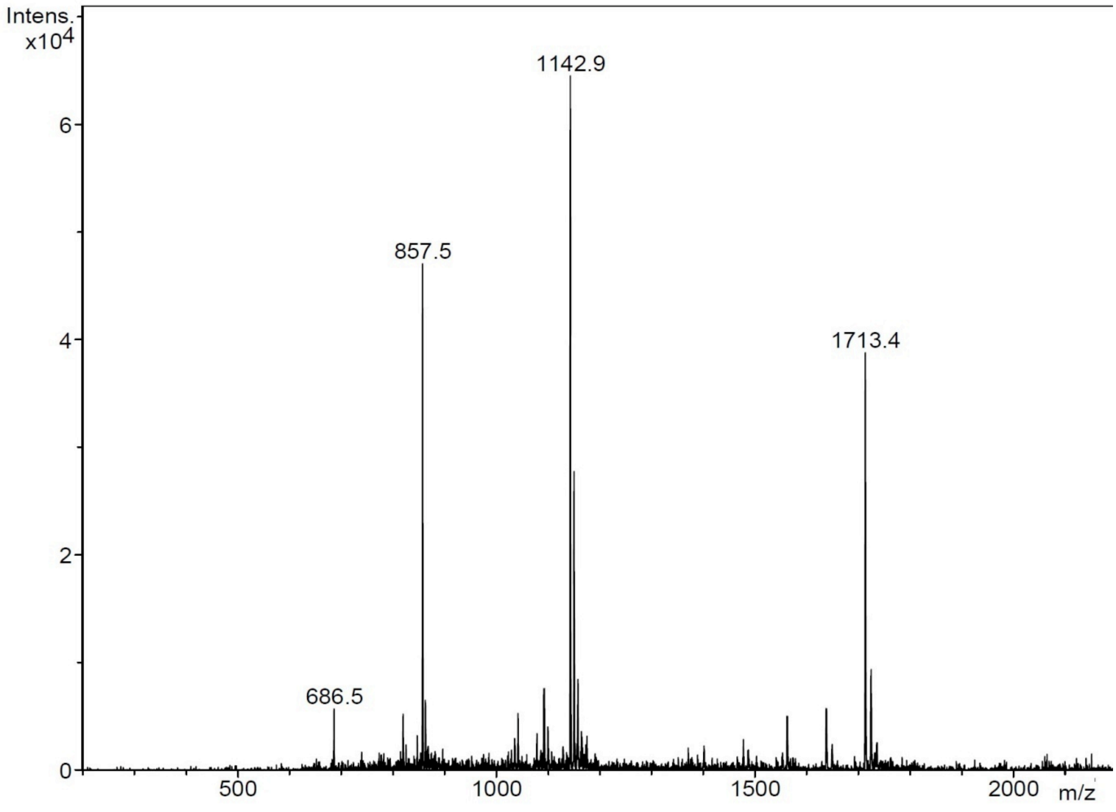

Ac-Lys-CGCGGTCCCAGACGCG-NH_2 (PNA 5)

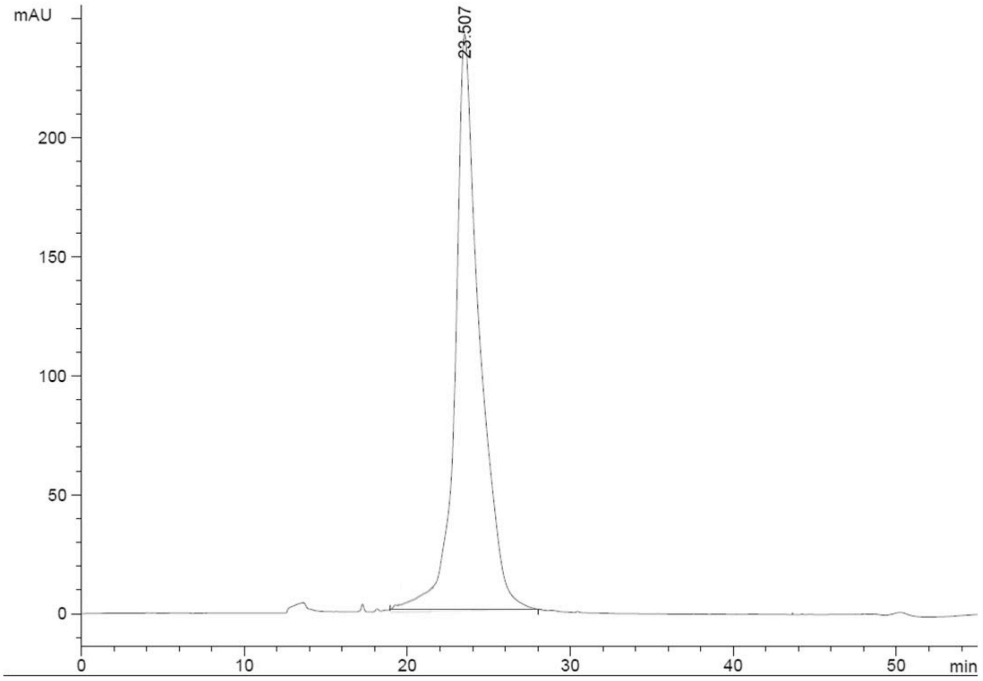

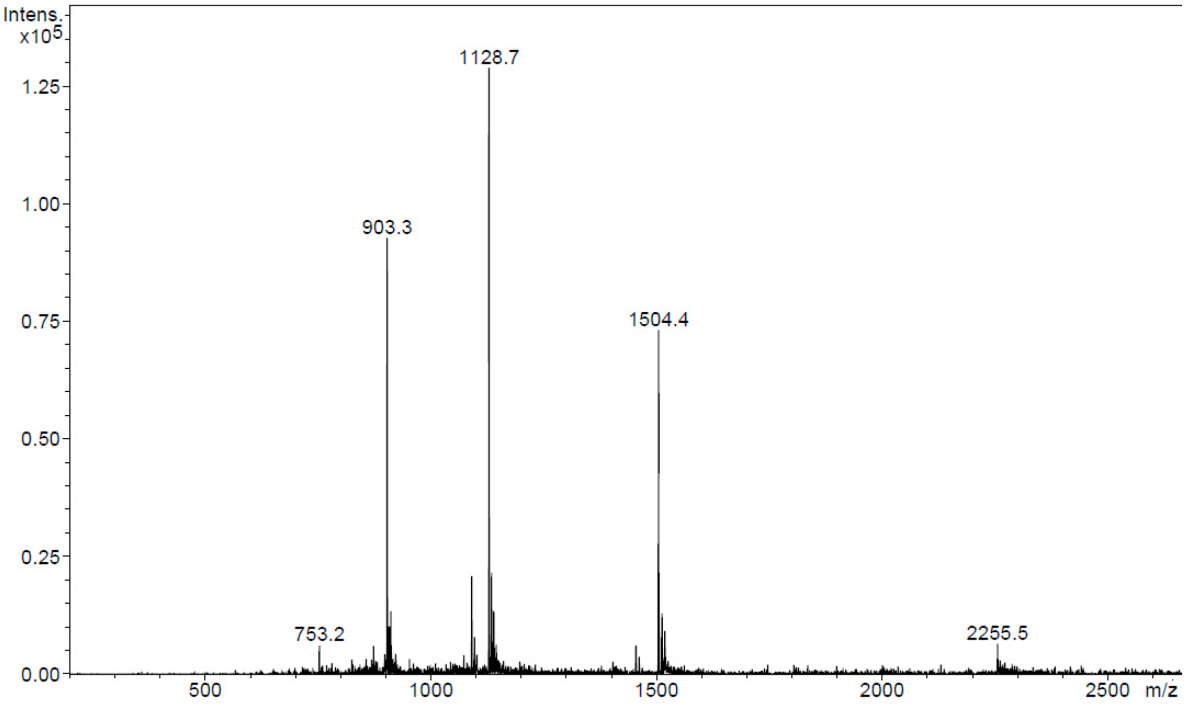

Ac-Lys-CGTCCCAGCG-NH_2 (PNA 6)

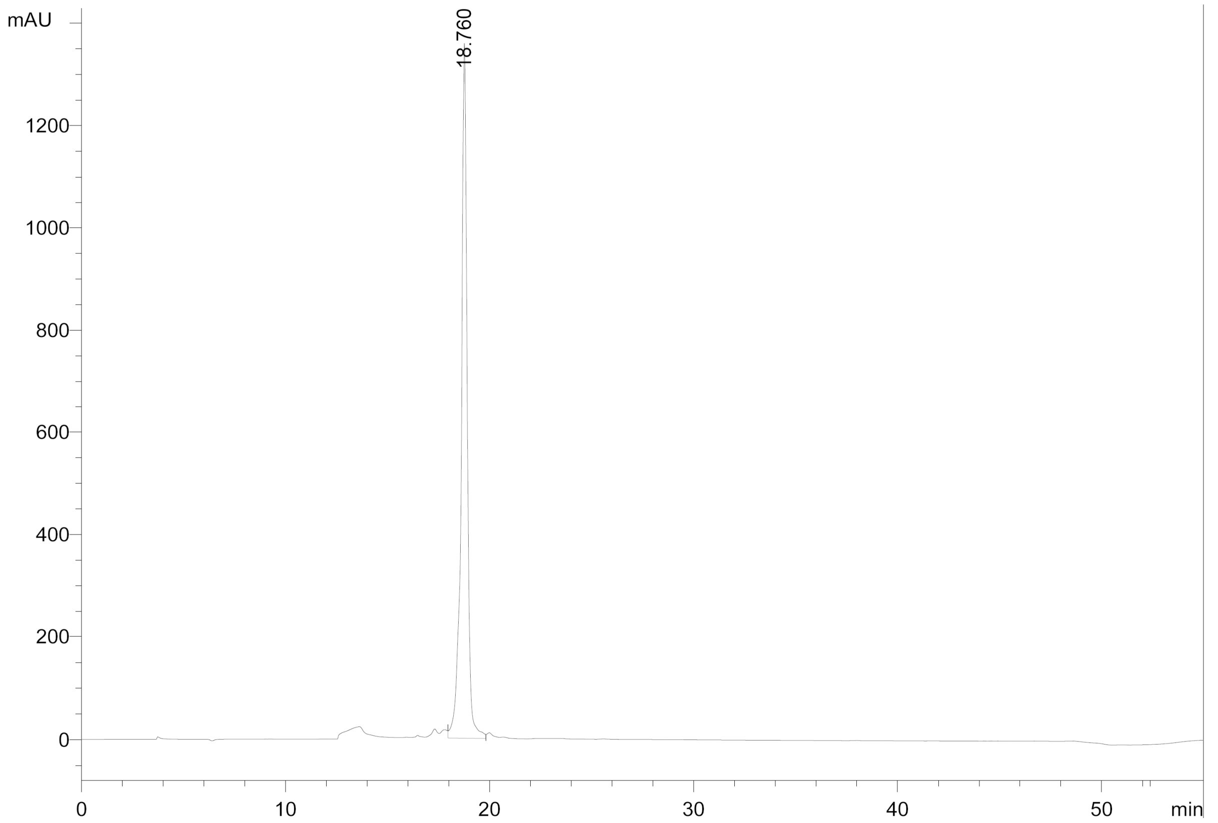

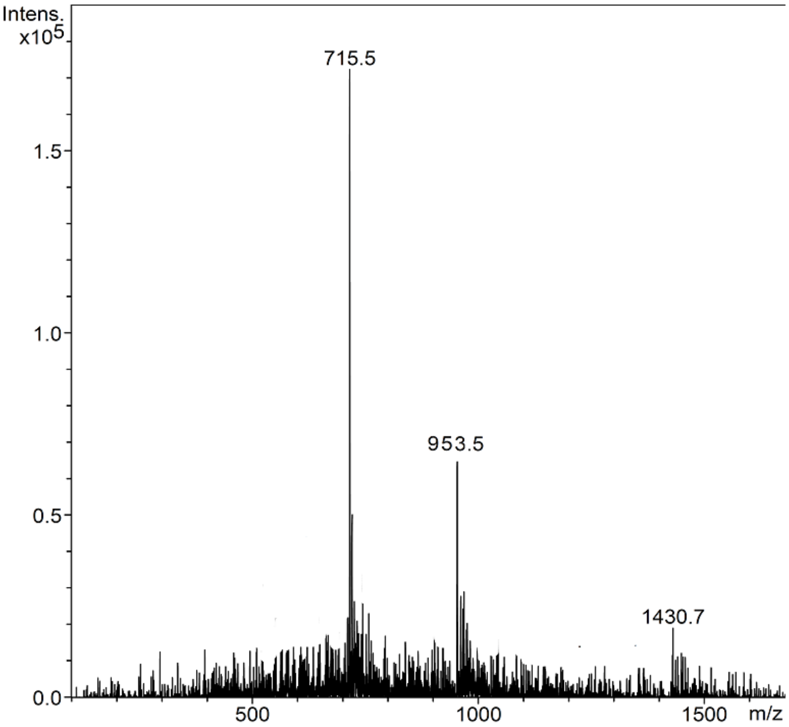

TPP-[PNA 1a]

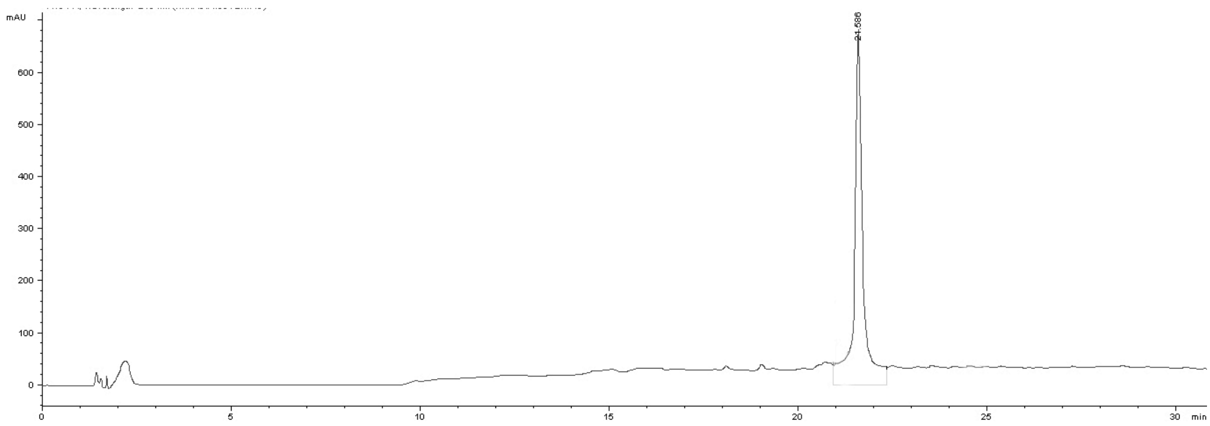

mAU
600
500
400
300
200
100
0
0
5
10
15
20
25
30
min

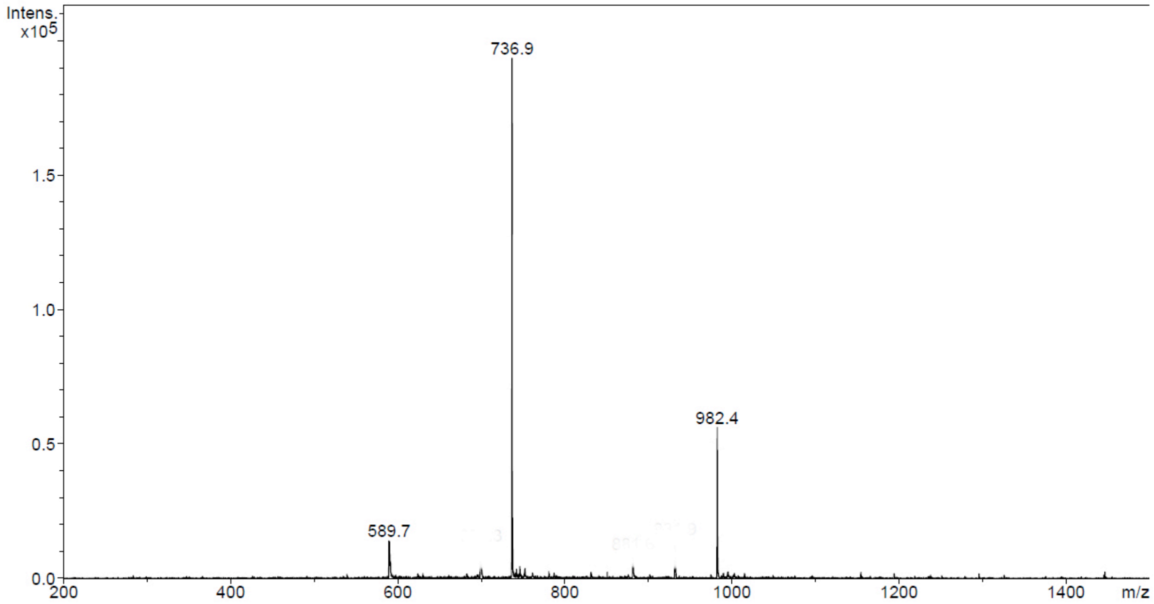

Intens.
x10^5
1.5
1.0
0.5
0.0
736.9
982.4
589.7
200
400
600
800
1000
1200
1400
m/z

TPP-[PNA 1b]

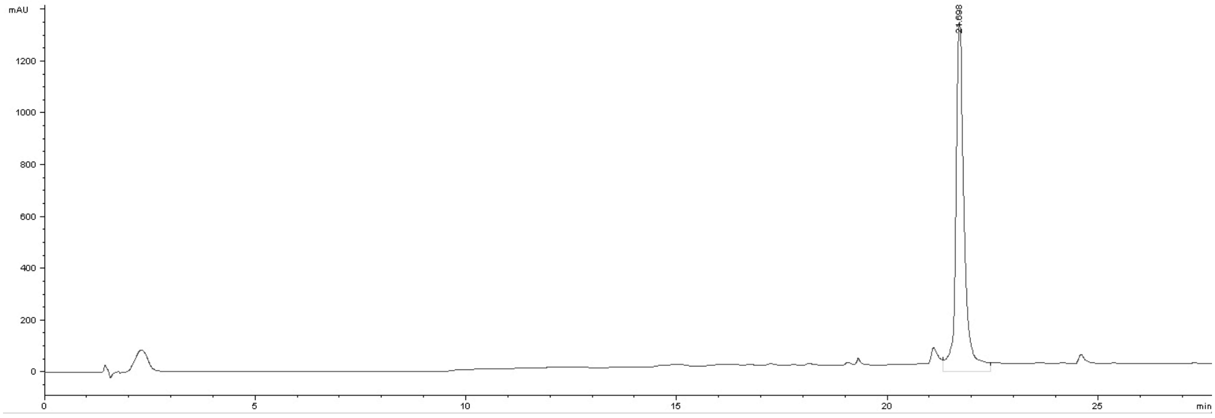
mAU
1200
1000
800
600
400
200
0
0
5
10
15
20
25
min

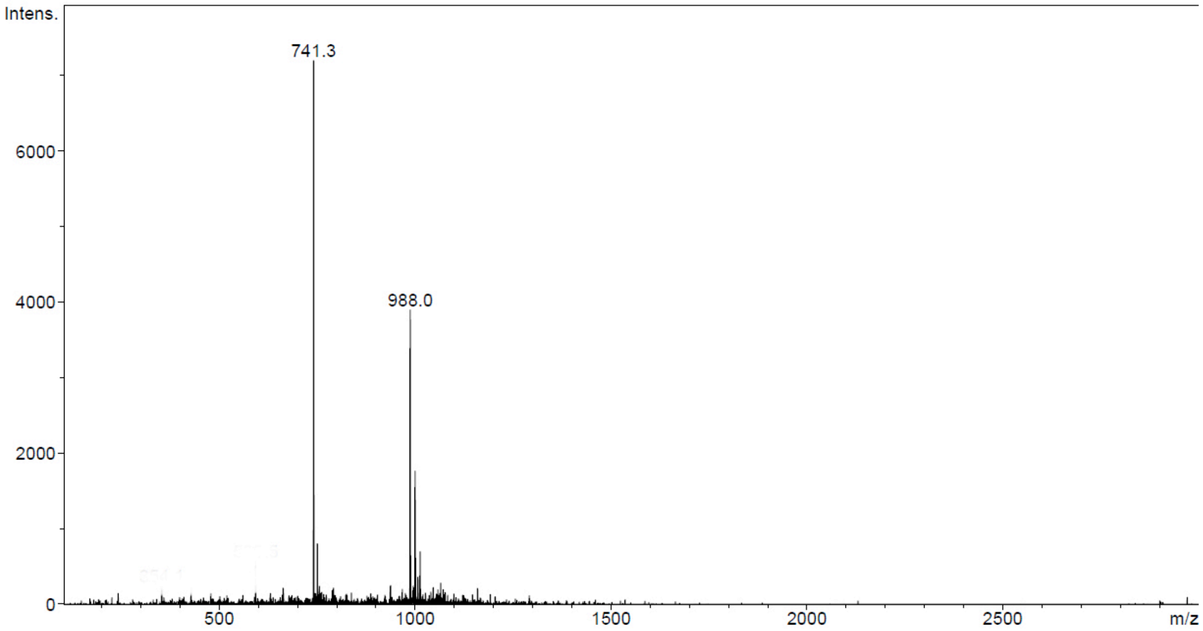
Intens.
741.3
988.0
6000
4000
2000
0
500
1000
1500
2000
2500
m/z

TPP-[PNA 1c]

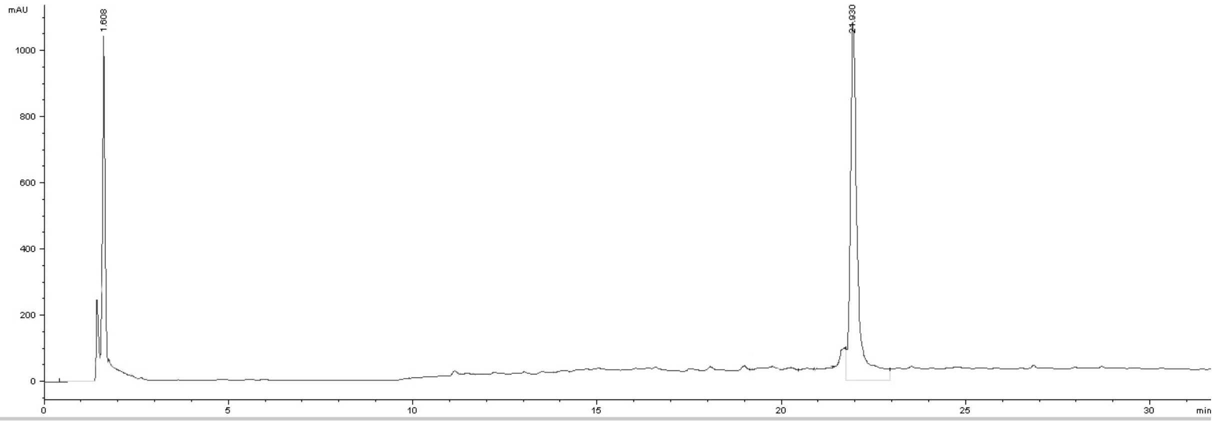

mAU
1.608
21.930
1000
800
600
400
200
0
0
5
10
15
20
25
30
min

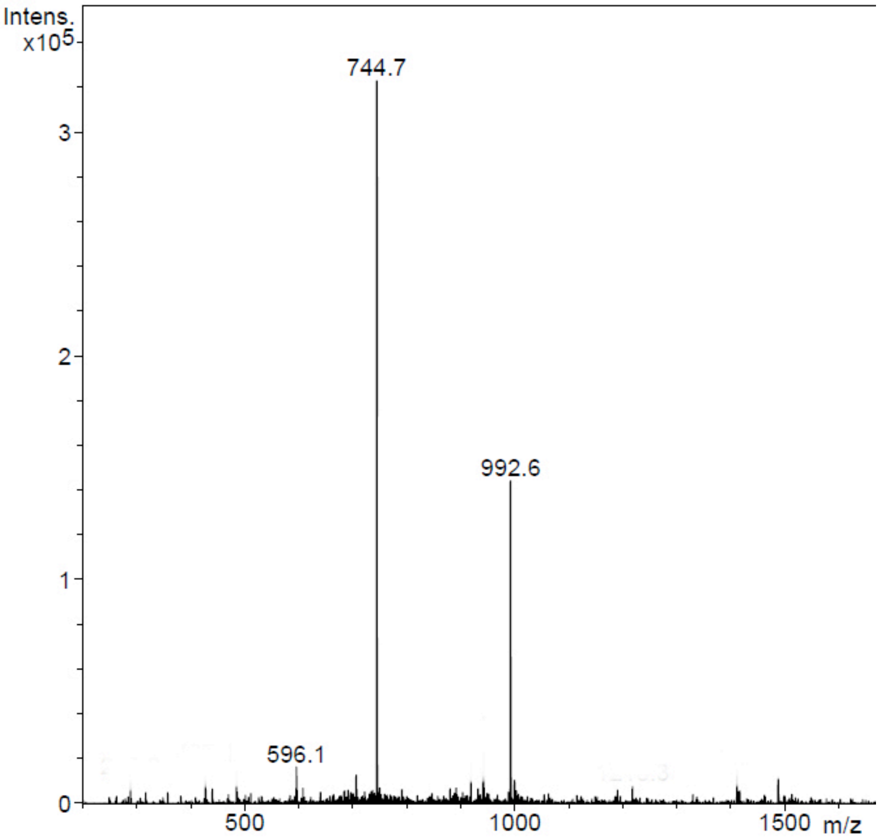

Intens.
x10^5
744.7
992.6
596.1
3
2
1
0
500
1000
1500
m/z

TPP-[PNA 2]

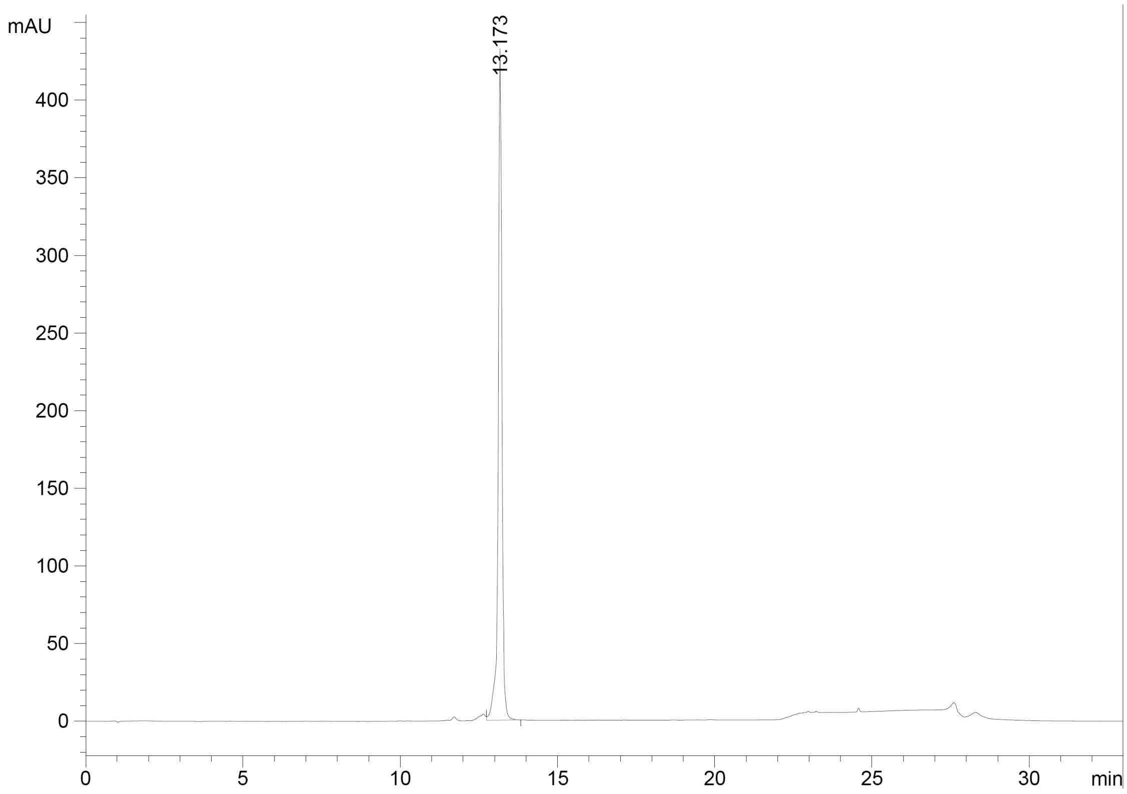
mAU
13.173
400
350
300
250
200
150
100
50
0
0
5
10
15
20
25
30
min

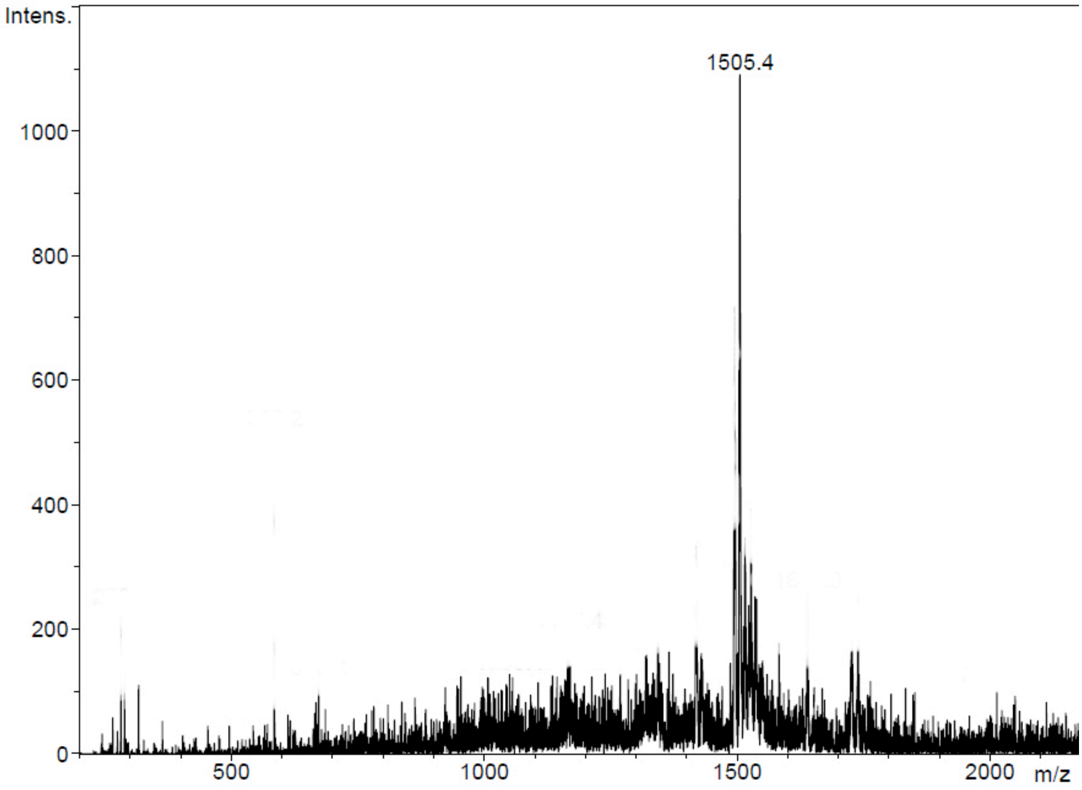
Intens.
1505.4
1000
800
600
400
200
0
500
1000
1500
2000
m/z

TPP-[PNA 3]

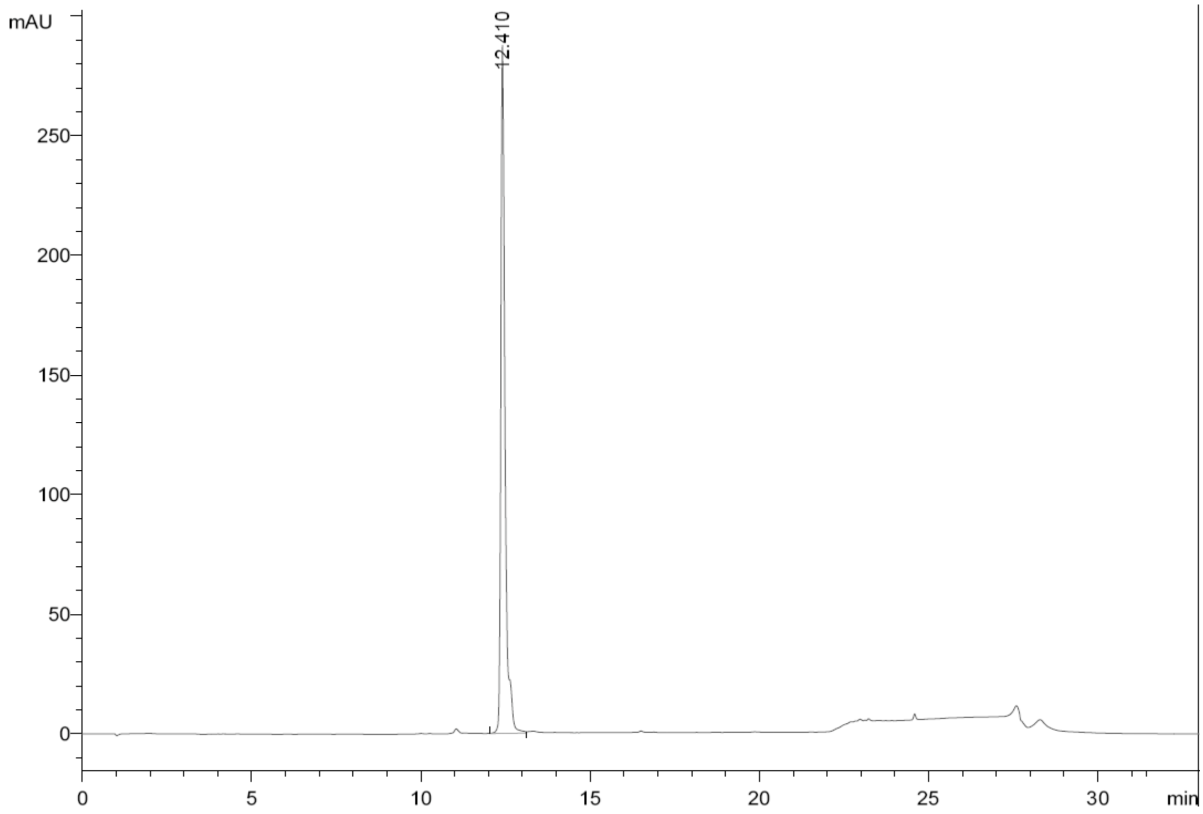

mAU
12.410
250
200
150
100
50
0
0
5
10
15
20
25
30
min

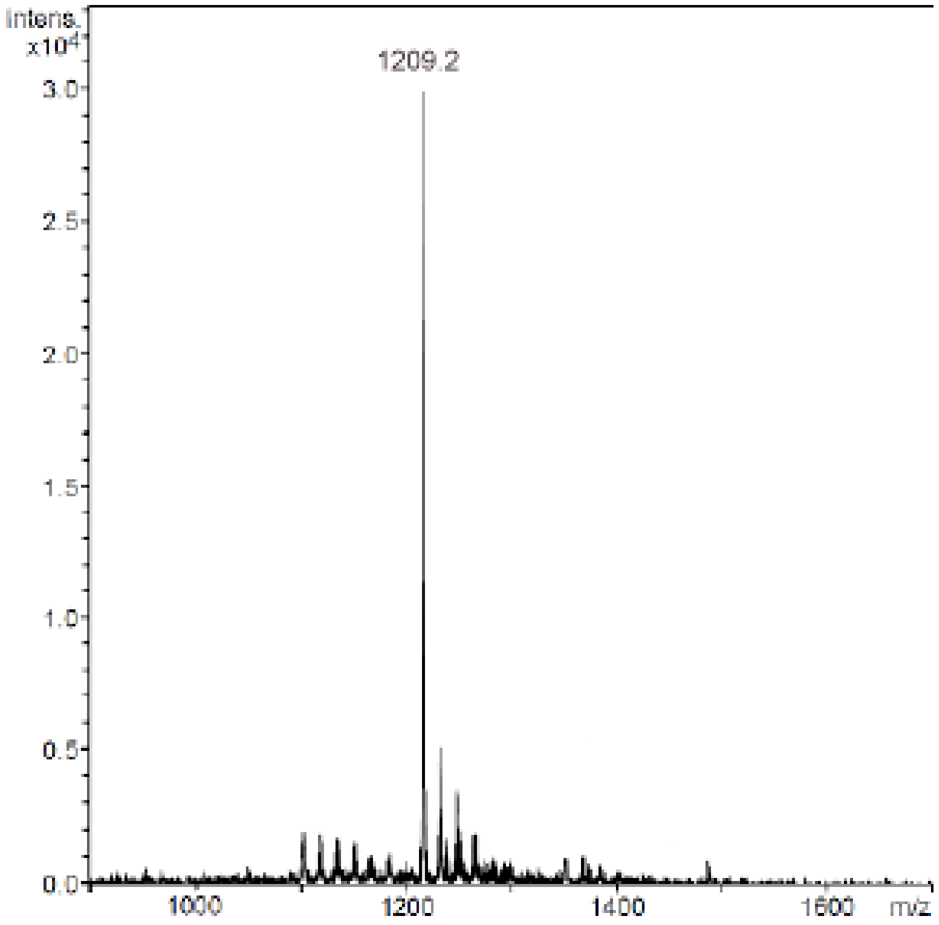

Intens.
x10^4
1209.2
3.0
2.5
2.0
1.5
1.0
0.5
0.0
1000
1200
1400
1600
m/z

TPP-[PNA 4]

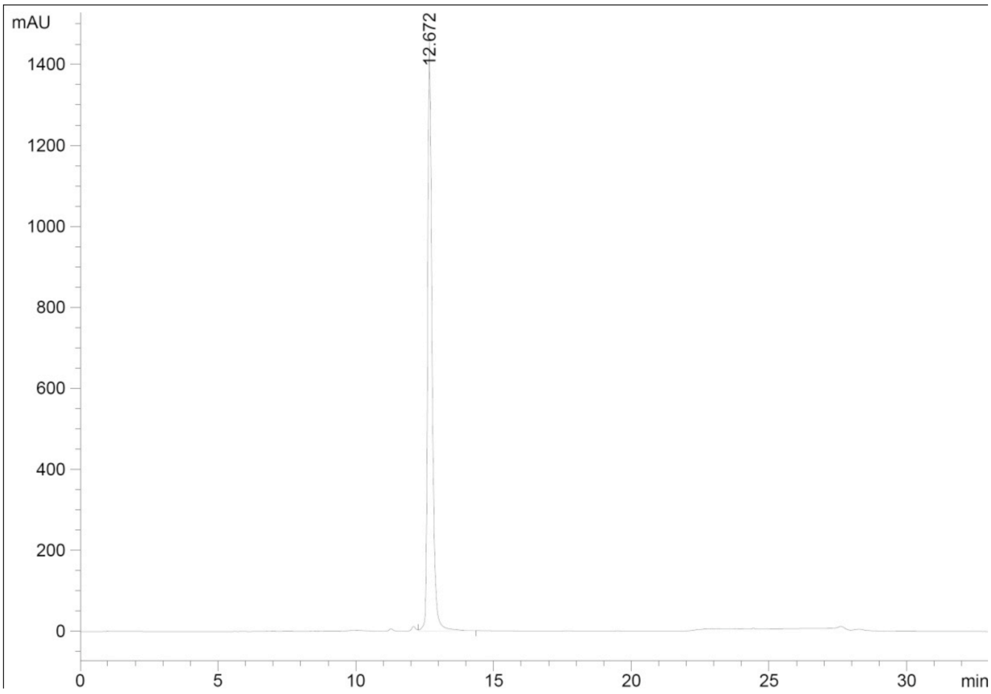

mAU
12.672
1400
1200
1000
800
600
400
200
0
0
5
10
15
20
25
30
min

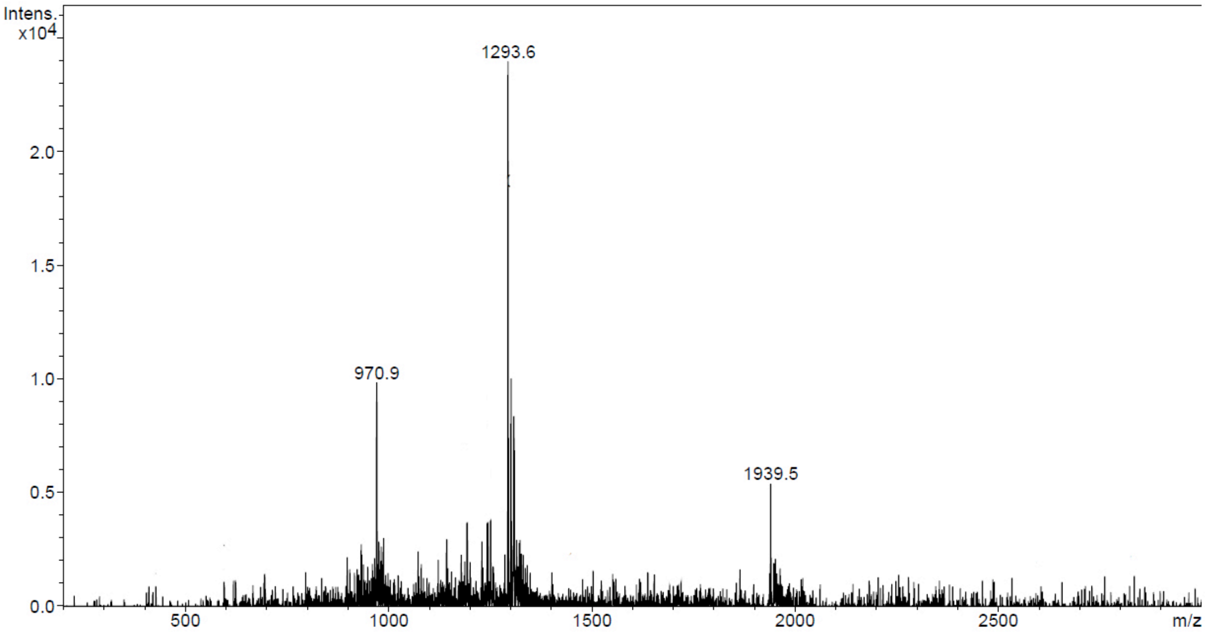

Intens.
x10^4
1293.6
2.0
1.5
1.0
970.9
0.5
1939.5
0.0
500
1000
1500
2000
2500
m/z

TPP-[PNA 5]

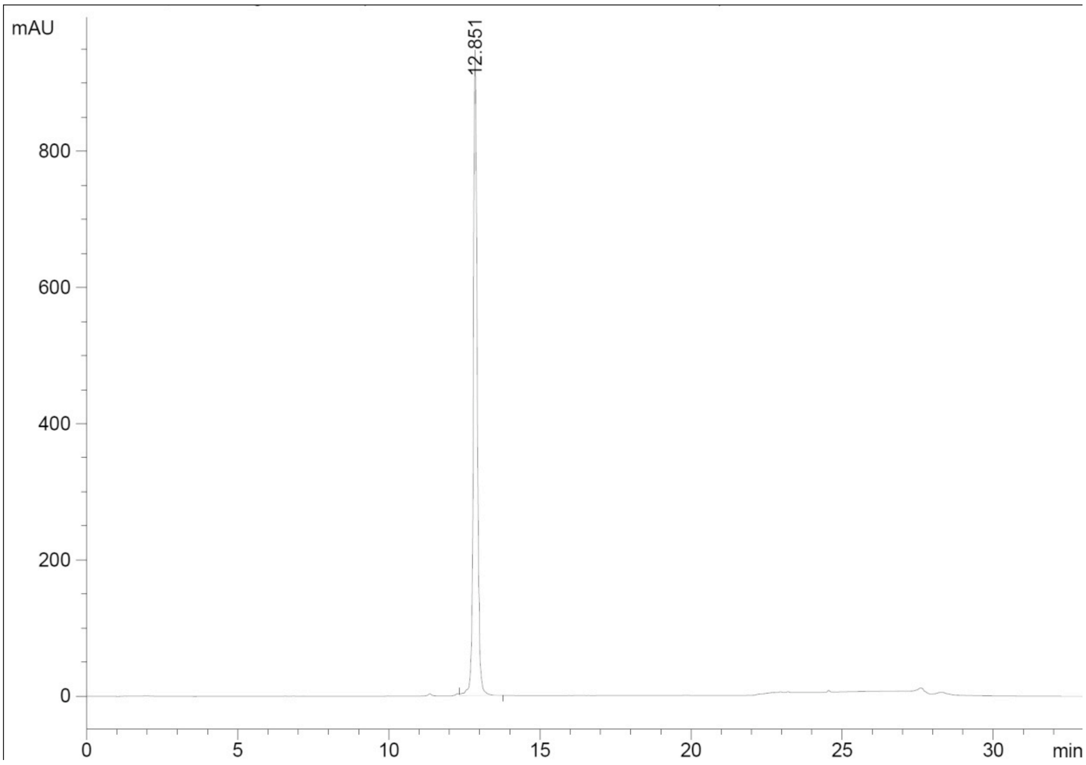

mAU
12.851
800
600
400
200
0
0
5
10
15
20
25
30
min

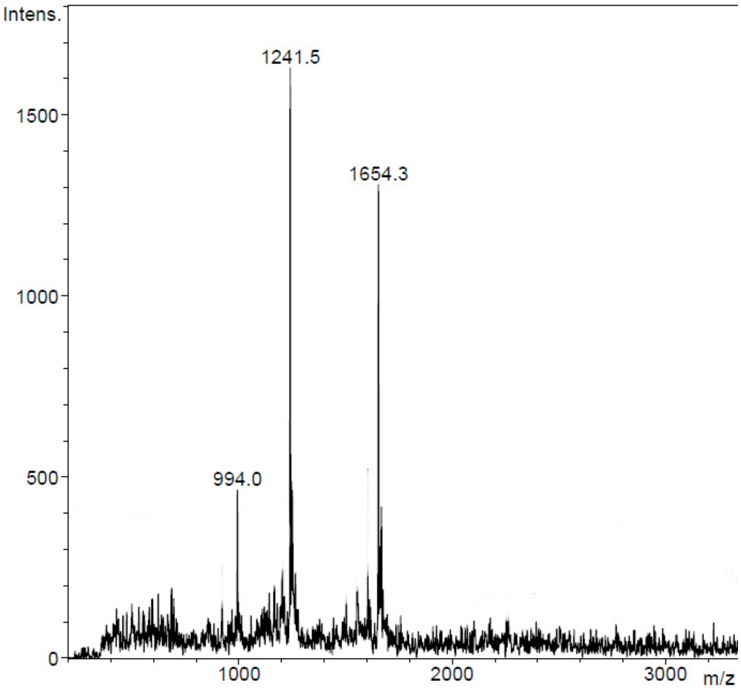

Intens.
1241.5
1654.3
994.0
1500
1000
500
0
1000
2000
3000
m/z

TPP-[PNA 6]

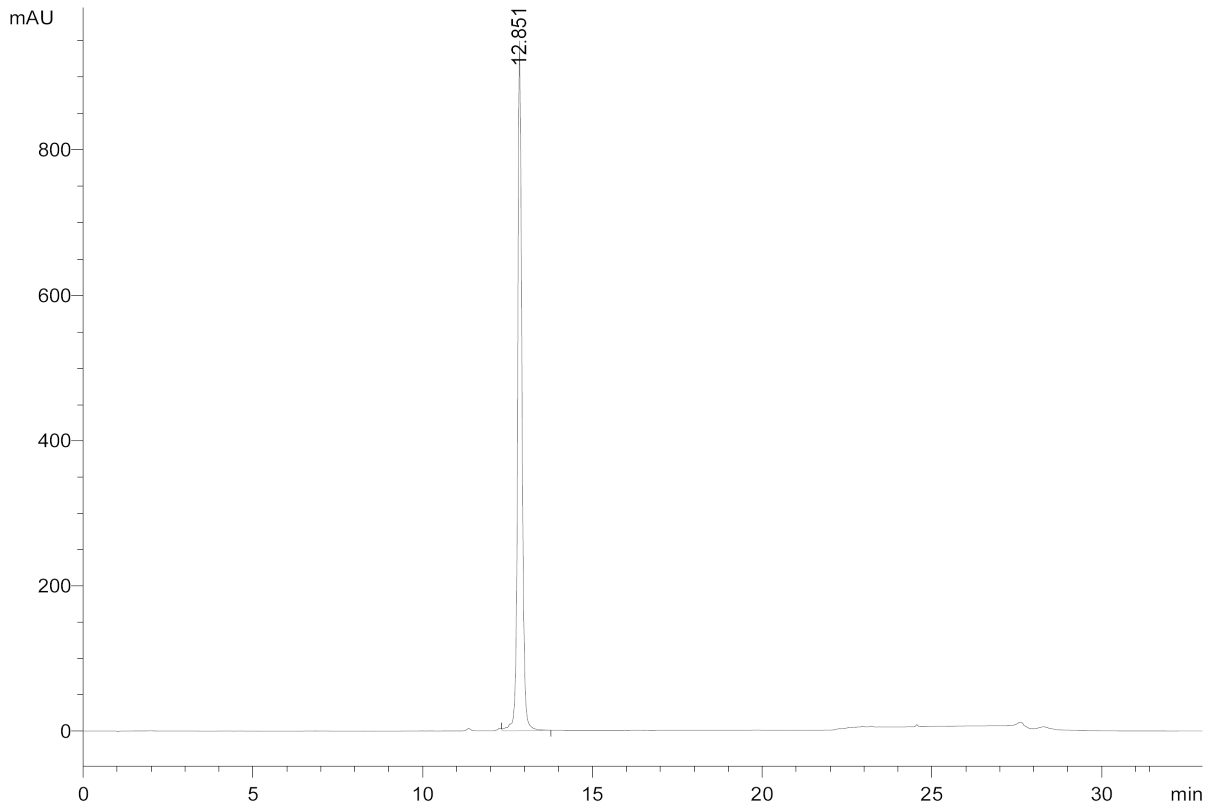

mAU
12.851
800
600
400
200
0
0
5
10
15
20
25
30
min

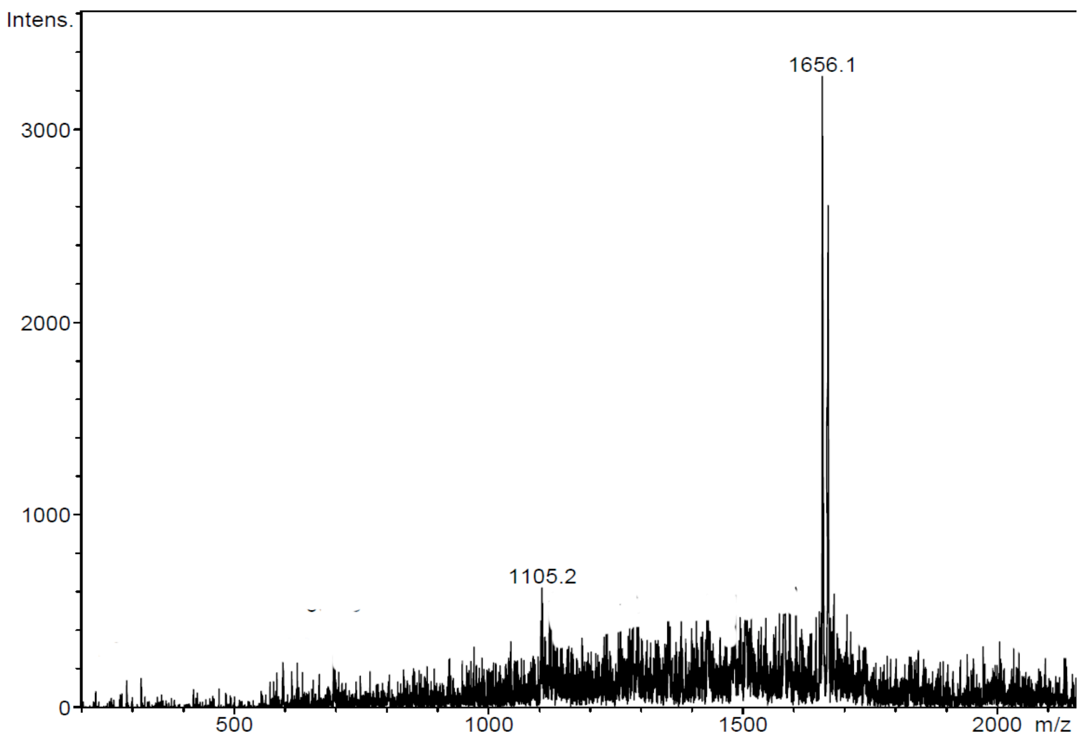

Intens.
1656.1
3000
2000
1000
1105.2
0
500
1000
1500
2000
m/z

B - Thermal denaturation followed by UV spectroscopy

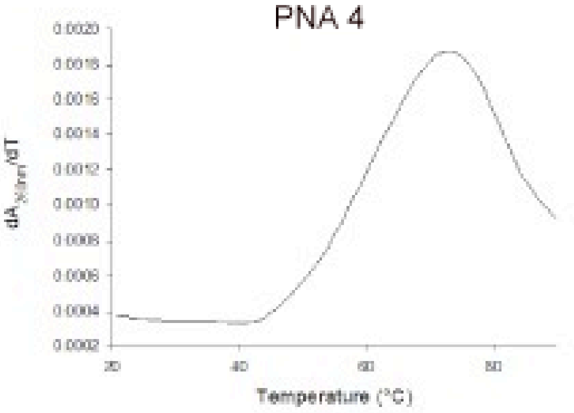

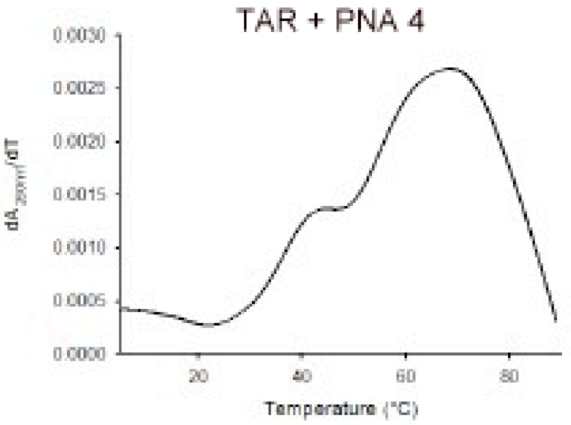

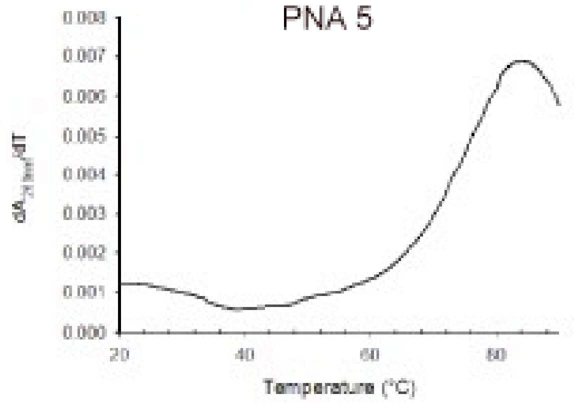

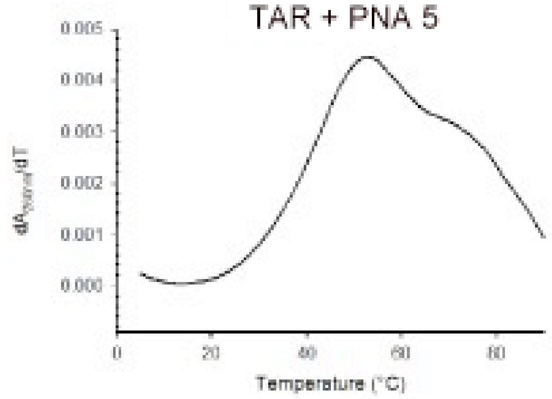

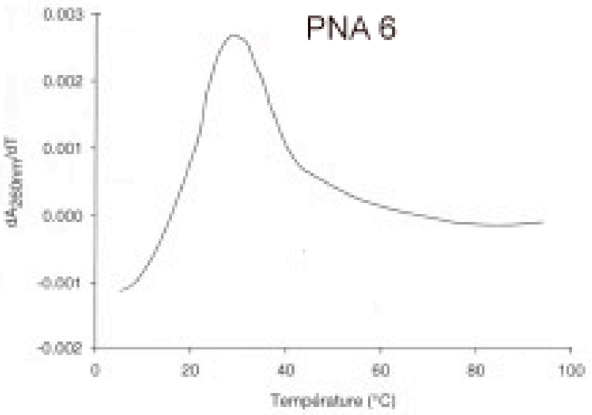

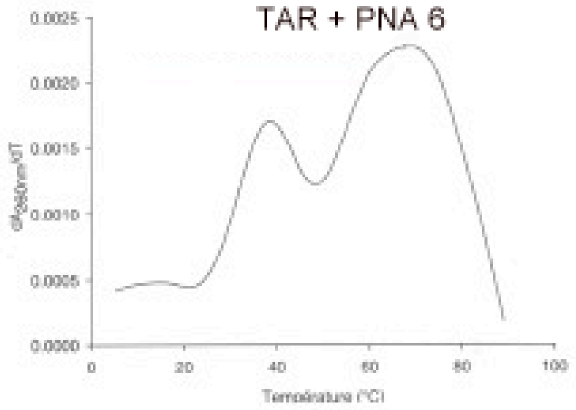

Supplement: Supplementary file 1 — Supporting Information Available: A) HPLC and ESI MS spectra of PNA 3-6 and TPP-[PNA 1a-c, 2-6] conjugates (p. S2-S13). B) UV-monitored thermal denaturation: First-derivative melting curves of PAA 4-6 and PAA 4-6/TAR complexes (p. S14). [file 591025.f1.pdf]
